# The role of lipids in exosome biology and intercellular communication: Function, analytics and applications

**DOI:** 10.1111/tra.12803

**Published:** 2021-06-11

**Authors:** Javier Donoso‐Quezada, Sergio Ayala‐Mar, José González‐Valdez

**Affiliations:** ^1^ Tecnologico de Monterrey School of Engineering and Science Monterrey Nuevo León Mexico

**Keywords:** exosome lipids, extracellular vesicles, lipid biomarkers, lipid trafficking, lipidomics

## Abstract

Exosomes are extracellular vesicles that in recent years have received special attention for their regulatory functions in numerous biological processes. Recent evidence suggests a correlation between the composition of exosomes in body fluids and the progression of some disorders, such as cancer, diabetes and neurodegenerative diseases. In consequence, numerous studies have been performed to evaluate the composition of these vesicles, aiming to develop new biomarkers for diagnosis and to find novel therapeutic targets. On their part, lipids represent one of the most important components of exosomes, with important structural and regulatory functions during exosome biogenesis, release, targeting and cellular uptake. Therefore, exosome lipidomics has emerged as an innovative discipline for the discovery of novel lipid species with biomedical applications. This review summarizes the current knowledge about exosome lipids and their roles in exosome biology and intercellular communication. Furthermore, it presents the state‐of‐the‐art analytical procedures used in exosome lipidomics while emphasizing how this emerging discipline is providing new insights for future applications of exosome lipids in biomedicine.

## Graphical abstract

Exosomes have gained attention due to their pivotal role in intercellular communication. Therefore, several exhaustive works have been performed to describe the main exosomal components (e.g., miRNA, proteins and peptides lipids) and their regulatory functions. However, despite the important advances in this field, research in exosome lipids is incipient and the current understanding of their regulatory functions is limited. This review presents the most recent advances in exosome lipids emphasizing their role in disease progression and future applications in biomedicine.

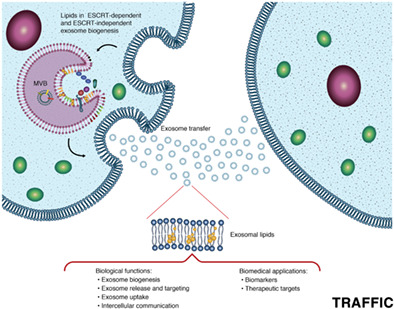

## 1 INTRODUCTION

Extracellular vesicles (EVs) are lipidic structures secreted by cells through diverse mechanisms as part of their natural physiological processes. Based on their origin, these structures can be defined as microvesicles, apoptotic bodies and exosomes. Microvesicles or ectosomes are EVs (50‐2000 nm) originated by direct outward budding of the cytoplasmatic membrane for intercellular communication purposes, meanwhile, apoptotic bodies are larger structures (500‐4000 nm) formed during apoptosis for the disintegration of the cellular content.^1^ On their part, exosomes are a particular subpopulation of EVs secreted by most cell types through the endocytic pathway.^2^ Exosomes originate from early endosomes by inward budding of the endosomal membrane, producing small structures named intraluminal vesicles (ILVs) within the multivesicular bodies (MVBs). The MVBs either fuse with the lysosome for the degradation of the ILVs or reach the cell membrane to release the ILVs as exosomes.^3^ Exosomes, like other EVs, are limited by a lipidic membrane, which encapsulates the cargo molecules in an inner aqueous core. In the particular case of exosomes, these cargo molecules are mainly peptides, small proteins and nucleic acids, such as mRNA or miRNA, all of them used by the cell to transmit signals to other cell populations, coordinate biological functions and maintain homeostasis.^4^ Despite its wide use in EVs reports, the application of the above‐mentioned terminology is misleading in the practice due to the current limitations to isolate a particular type of EVs in a pure form. Therefore, the International Society for Extracellular vesicles on the Minimal Information for Studies of Extracellular Vesicles 2018 (MISEV 2018) suggest the use of alternative terms such as “small EVs” (<200 nm) or “large EVs” (>200 nm).^5^

**FIGURE 1:**
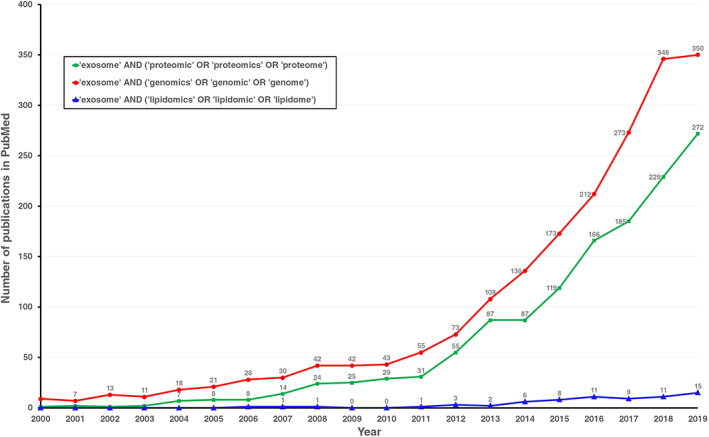
The number of publications between 2000 and 2019 in PubMed related to exosome genomics, proteomics, or lipidomics. The search terms were “exosome” and “proteomic”, “proteomics” or “proteome” (green); “exosome” and “genomics”, “genomic” or “genome” (red); “exosome” and “lipidomics”, “lipidomic” or “lipidome” (blue)

Lately, exosomal proteins and nucleic acids have received particular attention in several studies exploring the biological processes in which they are involved with therapeutic purposes.^6^, ^7^, ^8^ However, exosomal lipids represent other less‐explored bioactive molecules abundantly present in exosomes, not only as part of their structure but also exerting regulatory functions in receptor cells.^9^ Figure 1 shows the number of scientific publications indexed in PubMed related to exosome genomics, proteomics and lipidomics between 2000 and 2019. It should be noted that the scientific interest in all these three exosome‐related topics is constantly increasing year by year. However, in 2019, the research in exosome lipidomics represented <4.3% of the exosome genomics research, and approximately 5.5% of the exosome proteomics works, demonstrating that the scientific interest in exosome lipids and the processes in which they are involved are still incipient. Nevertheless, exosome lipidomics research has increased almost 15 times since 2006, indicating important advances in exosome‐lipid‐related technologies. Recently, these molecules have emerged as innovative biomarkers for several disorders and numerous models have been proposed to describe their biological effect and regulatory functions over specific cell populations.^9^, ^10^, ^11^, ^12^, ^13^ In this sense, recent lipidomic studies over exosomes derived from different cell types describe the lipidic composition of these EVs and propose alterations under pathological conditions to contribute to the current knowledge about the physiology of exosomal lipids.^14^, ^15^, ^16^

In this context and considering the increased interest in exosomal lipids as regulatory molecules and biomarkers observed during the last years, this review describes the most recent advances in exosome lipidomics and their applications, emphasizing the biological importance of exosomal lipids in producer cells and their regulatory function in receptor cells. We also analyze some critical challenges regarding the currently available methods for exosome lipidomic analysis, and some opportunities and future perspectives about the applications of this promising technology. It is important to note that according to the MISEV 2018,^5^ the term “extracellular vesicles” is preferred over exosomes since it is difficult to ensure that a particular subtype of EVs (i.e., exosomes) is present in a sample without contamination with other EVs populations. Therefore, in this review, the term exosome is used only to refer to small EVs (50‐150 nm) isolated by the commonly accepted methods (e.g., ultracentrifugation, ultrafiltration, precipitation, etc.), expressing cytosolic or transmembrane proteins specific for EVs (e.g., ALIX, syntenin, CD63, etc.), and reported as exosomes by the authors of the works cited in this review. Otherwise, the term EVs is used instead of exosomes.

## 2 EXOSOME LIPID COMPOSITION

Lipids are essential elements found in all cell types and abundantly distributed in EVs. Sphingomyelin, phospholipids, ganglioside GM3 and cholesterol are lipid classes commonly found in cell membranes and consequently in exosomes.^14^ However, the relative abundance of these lipids in exosomal membranes may vary depending on the producer cell type,^17^ the physiological stage of the producer cell,^16^ and the fate and function of the exosome.^18^ In this regard, several studies revealed that exosomes produced under different conditions modify their lipid and metabolite composition to modulate their biological function. For example, in vitro studies revealed that PC3 cells co‐cultured with the ether lipid precursor hexadecylglycerol secret exosomes enriched in ether lipids and with different protein composition, demonstrating the impact of external stimuli to modify the lipidic and nonlipidic exosome composition.^19^ Similar results were obtained with Huh7 cells co‐cultured with palmitate and LPC, which resulted in an enhanced release of EVs with pro‐inflammatory activity.^20^ Furthermore, it has also been found that exosomes derived from mesenchymal stem cells (MSC) cultured under priming conditions are packaged with lipids and other metabolites associated with the immunomodulatory properties of MSC, including macrophage polarization.^21^

The lipidic composition of exosomes derived from different sources and the enrichment of some lipid classes concerning producer cells has been extensively reported in several works.^17^, ^22^, ^23^ In this sense, B‐lymphocyte‐derived exosomes are enriched in cholesterol (CHOL) up to 3 times more when compared to the cell membrane.^24^ Apparently, CHOL starts to be accumulated in MVBs and this process appears to be essential for the formation of intraluminal vesicles, the precursors of exosomes.^22^ Similarly, sphingomyelin (SM) enrichment in exosome membranes has caused these EVs to be considered as a new type of SM domain. This enrichment could originate from plasma membrane lipid rafts, and also at the expense of phosphatidylcholine (PC) through the activity of the sphingomyelin synthase.^22^ Moreover, lipidomic studies in PC3 cells‐derived exosomes confirmed that the exosomal membrane is a highly ordered structure enriched in glycosphingolipids, which confers the exosomes the demonstrated stability they present in extracellular environments.^17^ This ordered distribution of lipids in the exosomal membrane could be responsible for several interactions during exosome formation, release and delivery to receptor cells, as discussed in the following subsections. It is important to note that the lipid distribution into exosomes and other EVs is a dynamic process that responds to several factors. For instance, significant variations in the lipidic composition of reticulocyte‐derived exosomes were found in response to the physiological changes in the cell during the maturation to erythrocytes, demonstrating that the sorting of lipids for exosome biogenesis adapts to the cell requirements.^16^

The distribution of lipids in the two leaflets of the lipid bilayer appears to be asymmetrical in the exosome membrane, with SM typically found in the outer leaflet and phosphatidylserine (PS) species in the inner leaflet.^25^ However, it has been reported that PS is externalized in apoptotic and malignant cells, acting as an “eat me” signal for macrophages in the immune system.^26^ Thus, exosomes and other EVs secreted by malignant cells also expose PS at the outer leaflet, opening novel perspectives for their potential use as exosomal biomarkers for cancer diagnosis.^27^ In this context, a PS‐targeted microfluidic device has been developed to isolate cancer‐derived exosomes from plasma, achieving 90% capture efficiency for cancer cell exosomes, and resulting in a promising tool to explore the role of exosomes and exosomal lipids in cancer progression.^28^ Conversely, other studies affirm that microvesicles and exosomes lack the membrane asymmetry found in producer cells because of the presence of a phospholipid scramblase in the exosome membrane, evidenced by the presence of PS and phosphatidylethanolamine (PE) in the outer leaflet.^29^

Besides, recent lipidomic studies revealed that some lipids are exclusively or preferentially distributed to certain types of EVs, suggesting the existence of various highly controlled processes involved in the biogenesis of EVs and cargo packaging in which lipids play an indispensable role.^30^ Some relevant studies regarding this differential distribution of lipids in EV subpopulations are presented in Table 1. It is also important to mention as well that the advances in purification methods have allowed the isolation of a novel and smaller vesicle that has been named “exomeres” (~35 nm).^15^ Despite structural similarities with exosomes, exomeres seem to differ in lipidic composition, presenting higher content of triglyceride (TG), ceramide (Cer) and lysophosphatidylglycerol (LPG) when compared to exosomes, as shown in Table 1. Hence, this differential lipidic composition of EVs allows lipids to be considered important markers to assess the purity of exosome preparations.^31^ Furthermore, recent studies reported that the lipid alterations in EVs isolated from pleural effusion of patients with pulmonary tuberculosis and lung cancer were different in small EVs regarding large EVs.^32^ These findings suggest that the differential distribution of lipids in EVs subpopulations could be used to identify more sensitive biomarkers contained in a particular type of EVs.

**TABLE 1:** Characteristic lipids classes in EVs subpopulations

EVs source	Isolation method	Analytical method	EVs populations	Enriched lipids	References
RBL‐2H3 cell line	Differential ultracentrifugation	Exosome‐donor cells labeled with fluorescent lipids and the isolated exosomes were classified according to their protein markers. The relative fluorescence was measured in each case.	MHC II‐enriched exosomes	PC	^33^
			CD81‐enriched exosomes	Cer	
B16‐F10	Differential ultracentrifugation followed by AF4 fractionation.	HPLC‐MS/MS	Small EVs	PC, LPE.	^15^
			Large EVs	PC, LPE	
			Exomeres	CerG2, CL, LPG, Cer, TG.	
MDA‐MB‐231			Small EVs	PI, PE, MG.	
			Large EVs	LPE, PS, Cer, CerG2.	
			Exomeres	MG, CerG2; Cer, LPG; LPC; PG; TG; CL.	
AsPC‐1			Small EVs	DAG, CerG1, CL, CerG2, SM, PI, PS, CerG3, PE, PC.	
			Large EVs	CerG1, PG, VerG2, SM, PI, PS, CerG3, PE, PC.	
			Exomeres	DAG, TG, LPC, Cer, CerG1, LPG, MG, PG.	
Human Urine	Differential ultracentrifugation	TLC and MALDI‐TOF‐MS	Exosomes	CerP, HexSph, LacCer, M(IP)2C, SHexCer, SHexSph.	^34^
			Microvesicles	PI‐Cer, MG.	
Human Mesenchymal Stem Cells	–	Affinity binding to specific molecules.	CTB‐binding EVs	GM1	^35^
			AV‐binding EVs	PS	
			ST‐binding EVs	Globotriaosylceramide	
3T3‐L1	Differential ultracentrifugation	HPLC‐Orbitrap‐MS	Small EVs	CHOL	^36^
			Large EVs	PS	
U87	Differential ultracentrifugation	Direct infusion‐MS/MS	Exosomes	Glycolipids, FFA.	^37^
			Microvesicles	Cer, SM.	
Huh7			Exosomes	Glycolipids, FFA, CL.	
			Microvesicles	Cer, SM.	
Mesenchymal Stem Cells			Exosomes	Glycolipids, FFA, CL.	
			Microvesicles	Cer, SM.	

Abbreviations: Cer, ceramide; CHOL, cholesterol; CL, cardiolipin; Cer, ceramide; CerG1‐3, glucosylceramides; CerP, Ceramide phosphates; DAG, diacylglycerol; FFA, free fatty acids; GM1, ganglioside; HexSph, Hexosyl sphingoid bases; LacCer, Lactosyl ceramides; LPC, lysophosphatidylcholine; LPE, lysophosphatidylethanolamine; LPG, lysophosphatidylglycerol; LPI, lysophosphatidylinositol; M(IP)2C, Mannosyl‐di‐PI‐ceramides; MG, monoglyceride; PC, phosphatidylcholine; PE, phosphatidylethanolamine; PG, phosphatidylglycerol; PI, phosphatidylinositol; PI‐Cer, PI‐ceramides; PS, phosphatidylserine; SM, sphingomyelin; SHexCer, Sulfatides hexosyl ceramide; SHexSph, Sulfatides hexosyl sphingoid bases; TG, triglyceride.

## 3 THE ROLE OF THE LIPIDS IN EXOSOME BIOGENESIS

Exosome biogenesis is a high‐regulated process in which the endosomal sorting complex required for the transport (ESCRT) plays an essential role, recruiting exosomal cargo components and inducing the formation of ILVs from the endosomal membrane.^30^ However, more recently, novel ESCRT‐independent mechanisms have received attention due to their capacity to induce EV formation in the absence of ESCRT machinery, one of them is the denominated lipid‐driven mechanism.^38^ Moreover, the enrichment of several lipid classes in exosomes and the differential lipidic composition of these EVs under different physiological conditions raises one question: what is the role of these lipids in the biology of exosomes? To answer this interrogation, this section focuses on the most relevant processes involved in exosome biogenesis in which lipids seem to play regulatory functions. Some important findings in this field are presented in Table 2. Moreover, we present a discussion about some possible future applications of this biogenic role of lipids in exosome‐related technologies.

**TABLE 2:** Role of some relevant lipids during exosome biogenesis

Lipid types	Process involved	Role in exosome biogenesis	Reference
Cholesterol	EVs formation, transport and release.	Provide adequate membrane conditions for budding by maintaining the equilibrium between liquid‐ordered and disordered domains. Interact with ORP1L and control the movement of endosomes along microtubules. Induce the fusion of MVBs with the cell membrane.	^39^, ^51^
Ceramide	EVs formation	Induce the negative curvature of the membrane.	^38^
Diacylglycerol	EVs formation	Recruit soluble proteins in the cell membrane. Interact with cytoskeletal proteins.	^52^
Ether lipids	EVs release	The fusion of MVBs with the cell membrane to release exosomes.	^19^
Phosphatidic acid	EVs formation	Responsible for several protein‐lipid interactions. Interaction with syntenin to recruit syndecan, CD63, and ALIX in the budding site. Induce membrane negative curvature.	^45^, ^46^
Phosphatidylinositol 3‐phosphate	EVs formation and cargo sorting.	Binding with ESCRT‐0 to recruit ESCRT‐I, ‐II and ‐III machinery in the membrane. Interaction with Hrs protein to begin the cargo sorting into endosomes.	^53^, ^54^
Bis(monoacyl‐glycero) phosphate	EVs formation and release.	Interaction with ALIX and HSP‐70. Fusogenic properties.	^55^, ^56^
Cardiolipin	EVs stabilization	Induce negative membrane curvature. Stabilize the small structure of the exosomes.	^37^
Phosphatidylinositol‐3,5‐biphosphate	EVs release	Regulation of lysosomal degradation of MVBs by fusion with lysosomes.	^57^
Sphingosine 1‐phosphate	Cargo sorting	Interaction with inhibitory G protein‐coupled S1P receptors in MVB membrane.	^44^

### 3.1 Cholesterol

**FIGURE 2:**
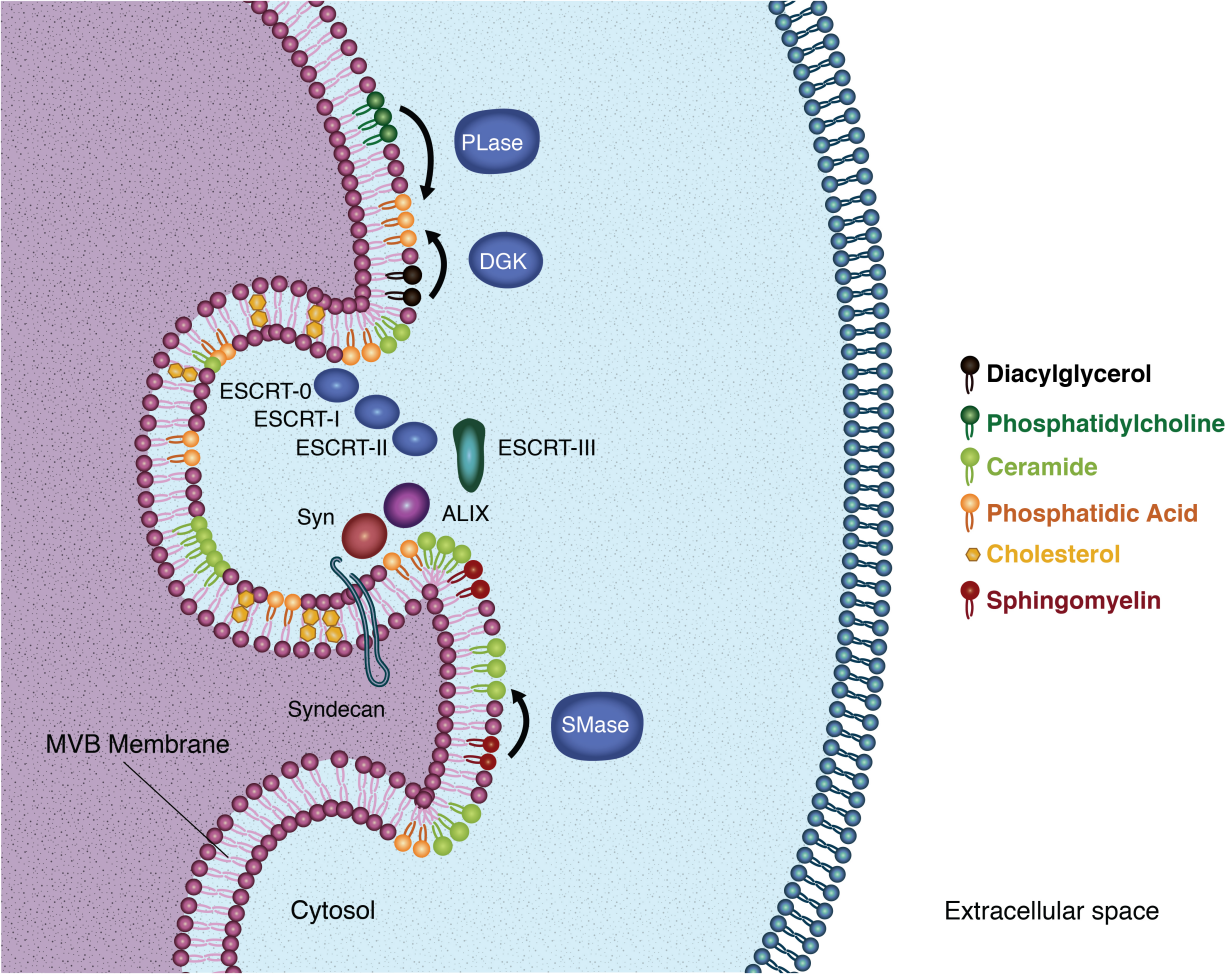
Lipids in exosome biogenesis. Membrane domains enriched in cholesterol appear to provide adequate conditions for the recruitment of ESCRT machinery in MVBs. Ceramide and phosphatidic acid are cone‐shaped lipids that seem to induce spontaneous curvature of the MVBs membrane in an ESCRT‐independent manner. Ceramide is produced from sphingomyelin through the activity of the sphingomyelinases (SMase). On their part, phosphatidic acid is produced from phosphatidylcholine and diacylglycerol through the activity of the phospholipases (PLase) and diacylglycerol kinases (DGK), respectively. Furthermore, phosphatidic acid seems to interact with syndecan to enhance the recruitment of syntenin (Syn), ALIX and the ESCRT machinery

The ESCRT machinery plays a fundamental role in the formation of MVBs and the packaging of cargo components into exosomes.^30^ In vitro studies revealed that the ESCRT machinery induces the formation of ordered membrane microdomains in a CHOL‐dependent manner, suggesting that CHOL content within endosomal membranes may provide adequate conditions for exosome formation, as shown in Figure 2.^39^

The identification of novel molecules with regulatory functions over the biogenic processes of exosomes may represent the opportunity to discover novel therapeutic targets to control the secretion of these EVs, and consequently, to regulate the activation of exosome‐related signaling pathways associated with pathological conditions such as metastasis and inflammation. For example, the hepatoma cell line Huh‐7 treated with cholesterol resulted in a reduced number of MVBs co‐localized with lysosomes and an increased secretion of exosomes with the capacity to induce M1 polarization in THP‐1 monocytes.^40^ In this sense, the use of statins was reported to reduce the exosome release in BEAS‐2B and THP‐1 cells owing to its cholesterol‐lowering effect,^41^ opening novel perspectives regarding the use of statins as therapeutic agents to control exosome production in target cells.

### 3.2 Sphingolipids

Ceramide is one of the most important lipids in exosome biogenesis because of its apparent capacity to trigger ESCRT‐independent processes and induce spontaneous membrane invagination (Figure 2).^42^ Ceramide is synthesized from SM after removal of a phosphocholine moiety by sphingomyelinases, and the spontaneous budding of ceramide‐containing membranes is attributed to its cone‐shaped structure, which facilitates the negative curvature of the membrane.^38^ In vitro experiments revealed the capacity of sphingomyelinases by themselves to induce membrane budding and vesicle formation in synthetic membranes containing SM after ceramide synthesis.^43^ Therefore, the use of exogenous sphingomyelinases may represent an alternative to enhance the in vitro production of exosomes from cell lines of interest for scientific or therapeutic purposes.

Similarly, ESCRT‐independent cargo sorting in exosomes occurs in the cells through the constitutive activation of inhibitory G protein‐coupled sphingosine 1‐phosphate (S1P) receptors by a constant supply of S1P, representing another lipid‐regulated mechanism for the maturation of exosomal MVBs.^44^

### 3.3 Phospholipids

Like ceramide, the phosphatidic acid (PA) is the simplest phospholipid with a small headgroup and cone‐shaped structure that confers PA the capacity to induce spontaneous negative curvature in lipidic membranes (Figure 2).^45^, ^46^ Moreover, the physicochemical properties of PA are given in part by its headgroup, allowing protein‐lipid interactions between PA and the lysine and arginine residues of proteins.^47^ Hence, PA is reported to interact with syntenin triggering the recruitment of syndecan, CD63 and ALIX in the membrane, stimulating the budding process of nascent ILVs.^48^ Furthermore, it is proposed that sphingomyelinases interact with PA to enhance ceramide production and promote the ILVs budding in an ESCRT‐independent way.^49^ The synthesis of PA in the cells is regulated by the activity of phospholipases,^50^ therefore the use of exogenous phospholipases could be explored to increase the production of exosomes in vitro and to support the development of exosome‐related technologies. Other phospholipids that appear to play important regulatory functions during exosome biogenesis include phosphatidylinositol 3‐phosphate and phosphatidylinositol 3,5‐biphosphate, which seem to regulate the EVs formation, release and cargo sorting, as shown in Table 2.

## 4 EXOSOMAL LIPIDS AND CELL‐TO‐CELL COMMUNICATION

Exosomes act as nanocarriers of bioactive lipids between cells to regulate specific biological processes. However, their activity is not limited to transport lipids from one cell to another, but also to produce bioactive lipids from other lipidic molecules through the activity of exosomal enzymes packaged into these EVs during their biogenesis.^58^ In this sense, this section focuses both on the role of exosomes as lipidic particles as well as functional units for lipid transformation.

### 4.1 Lipid transformations in exosomes

Exosomes contain all three A2 phospholipases classes (PLA2); the calcium‐dependent PLA2 (cPLA2), the calcium‐independent PLA2 (iPLA2), and the secreted PLA2 (sPLA2). These enzymes hydrolyze glycerophospholipids to produce arachidonic acid (AA) and other free fatty acids.^59^ AA can be further processed by the 5‐lipoxygenase to release a set of oxidized eicosanoids named leukotrienes such as LTB4, involved in the inflammation process, and the angiogenesis‐promoting LTC4 and LTD4.^60^ On their part, the exosomal constitutive and inducible cyclooxygenases (COX1 and COX2, respectively) promote the transformation of AA into prostaglandin PGH2, which is subsequently transformed in the pro‐inflammatory prostaglandin E2 (PGE2) by PGE synthase or in the anti‐inflammatory and tumor‐suppressing 15‐deoxy‐prostaglandin J2.^59^ In addition, the exosomal phospholipase D (PLD) hydrolyzes PC into PA, which can act as a second messenger to interact with RAF kinases and mTOR with mitogenic effects.^61^

### 4.2 Interactions of exosomal lipids with recipient cells

Exosomal lipids appear to be involved both in the fate and internalization of exosomal material into recipient cells. In this sense, the fate of exosomes depends on the interaction of PS and lysophosphatidylcholine (LPC) with their respective receptors in target cells. PS binds the immunomodulatory TIM‐1 and TIM‐4 receptors. PS can bind simultaneously both receptors in different cells, acting as a bond between T‐cells and antigen‐presenting cells, therefore facilitating their interaction and antigen presentation.^26^ On their part, LPC is produced in exosomes through the activity of iPLA2 and cPLA2 and interacts with the G protein‐coupled receptor G2A.^59^, ^62^ LPC acts as a chemoattractant for T‐cells and prompts the maturation of immune cells.^63^

On the other hand, internalized exosomes located into recipient‐late endosomes release their cargo in the cytosol by fusion of the exosomal membrane with the endosome. For that, fusogenic lipids such as PA and Bis(monoacylglycero)phosphate (BMP) are required in the exosomal membrane. PA induces the exclusion of water molecules on the polar‐head group of phospholipids, making membrane fusion possible.^64^ BMP is produced in the exosome through the activity of PLD and PLA2 and triggers the fusion of membranes in acidic conditions.^65^

### 4.3 Exosome lipids and disease

The capacity of exosomal lipids and their derivatives to interact with recipient cells makes these molecules important mediators of disease progression. For example, it was found that exosomes isolated from bronchoalveolar lavage fluid of asthmatic patients contained significantly lower phosphatidylglycerol (PG), ceramides and ceramide phosphates, resulting in altered airway surfactant compositions, impaired immune signaling, and consequently, reduced lung function.^66^ Moreover, exosome‐encapsulated mitochondria were found in the human‐bronchoalveolar fluid, suggesting that this mechanism is used to transfer this organelle from myeloid‐derived regulatory cells to T‐cells and induce pro‐inflammatory responses, especially in asthmatic patients.^67^ These findings are congruent with those reported by Haraszti et al.,^37^ who report enrichment of cardiolipins (i.e., lipids believed to exclusively exist in the inner mitochondrial membrane) in exosomes isolated from Huh‐7 and MSC.

Furthermore, the myoblast cells C2C12 exposed to palmitate produced palmitate‐enriched exosomes with the capability to induce myoblast proliferation and to alter the expression of genes involved in cell cycle and muscle differentiation. Besides, these exosomes were able to be incorporated in various tissues in vivo, including the pancreas and liver, transferring by this way the deleterious effect of palm oil between muscle cells and other tissues.^68^

In the brain, as an organ with one of the highest lipid concentrations,^69^ not only exosomal lipids exert regulatory functions, but also the lipid‐processing enzymes packaged into these EVs. It has been demonstrated that the cerebrospinal fluid of Multiple Sclerosis patients contains acid sphingomyelinase‐enriched exosomes, which transform the SM in ceramides, inducing axonal damage, and mitochondrial dysfunction in this disease.^70^

A different mechanism for exosomal lipid‐mediated regulation has been proposed in the human pancreatic tumoral SOJ6 cell line, opening new perspectives for exosome‐based cancer treatment. In this regard, Beloribi et al^18^ demonstrated that synthetic lipidic particles with a lipidic composition like SOJ6‐ derived exosomes induced mitochondria‐dependent apoptosis by inhibiting the Notch‐1 pathway through the modification of the lipidic microenvironment of the cell membrane. This process occurs because of the sensitivity of the γ‐secretase complex to the lipid microenvironment of the membrane.^71^ However, this effect seems to be cell‐type specific. In Mia‐paCa‐2 cells, these synthetic lipidic particles also induce a downregulation of the Notch‐1 pathway but with no alterations in the downstream targets such as the Bax to Bcl‐2 ratio.^72^ In this case, the exosome lipids could contribute to tumor progression and drug resistance through the activation of the Akt survival pathway.

Similarly, lipids in different EV populations seem to regulate signaling pathways related to the pathology of metabolic disorders. In small EVs isolated from adipose tissue of mice, Crewe et al.^73^ found an enrichment of ceramides and sphingolipids with relevant implications in the activation of nutrient stress responses. Likewise, Flaherty et al.^74^ reported a novel exosome‐mediated mechanism for lipid release in adipocytes. They found that adipocytes‐derived exosomes represent an important source of lipids for local macrophages with the capability to induce in vitro differentiation of bone marrow precursors into adipose tissue macrophage‐like cells. On the other hand, EVs secreted by hepatocytes appear to change their lipidic composition in response to a lipotoxic environment in vitro.^75^ In this sense, mice hepatocytes treated with palmitic acid produce EVs enriched in S1P with chemoattractive properties to macrophages.^76^ Besides, Hirsova et al.^20^ found increased production of EVs in Huh‐7 cells treated with palmitate and or lysophosphatidylcholine. These EVs were able to activate an inflammatory phenotype in macrophages.

Other processes regulated by exosome lipidic components that may be involved in disease progression include the platelet aggregation and activation induced by the thromboxane synthesized in the exosomes from AA via COX1, COX2 and thromboxane synthase activity^77^; the expansion of myeloid‐derived suppressor cells and the consequent tumor growth incited by the exosomal PGE2^78^; the recruitment of Th17 cells in the intestine and subsequent establishment of tumors in colon induced by S1P and PGE2‐containing exosomes^79^; the regulation of eosinophils activation by LPC and prostaglandin D2‐enriched exosomes secreted by *Schistosoma mansoni* during parasitic infections^80^; the accumulation of cholesterol in cells of the atherosclerotic plaque, induced by PS‐mediated uptake of CD4+ T Cell‐derived exosomes^9^; among others. This evidence emphasizes the importance of exosome lipidomic studies to improve the current knowledge regarding the mechanisms involved in disease progression, and the later development of novel therapeutic strategies based on exosomal components.

## 5 NOVEL ANALYTICAL PROCEDURES FOR EXOSOMAL LIPID ANALYSIS

Despite the significant advances achieved in recent years, most of the functional roles of lipids in the regulation of cellular homeostasis remain to be elucidated.^81^ In this context, the improvement of analytical methods has allowed the evaluation of the complete set of lipids in organisms, tissues, cells, or specific organelles, assessing crucial aspects such as the quantification of their abundances and the analysis of the interactions with other molecules, leading to the field of lipidomics.^82^ However, the structural lipid diversity and mixture complexity of lipid preparations arise significant challenges in lipidomic studies.^81^ Thus, advances in the analytical methods attempt to improve their ability to reach a complete analytical coverage and to estimate accurate concentrations of each lipid in a mixture. Here, we discuss some relevant aspects regarding the current methods applied in lipidomic studies, emphasizing their applications in exosome‐related technologies.

### 5.1 Advances in analytical procedures

Advances in mass spectrometry (MS) have made this method the dominating platform in the lipidomic analysis.^83^ On its part, nuclear magnetic resonance (NMR) has become a less‐used system limited by its lower sensitivity, the presence of overlapping signals, and the low natural abundance of ^13^C for ^13^CNMR.^84^, ^85^ However, despite limitations, NMR is a powerful analytical method that permits the visualization of single atoms and molecules for lipid identification, with important applications in biomedicine^86^, ^87^, ^88^ and food science.^89^, ^90^, ^91^ Lipid analysis on MS‐based platforms can be classified into three main categories: direct infusion‐based “shotgun” analysis, liquid chromatography (LC)‐MS systems and gas chromatography (GC)‐MS platforms, each one with unique properties.^83^

Shotgun lipidomics has emerged as a technique for lipid analysis without chromatographic separation. The continuous sample introduction with electrospray ionization (ESI) coupled with tandem MS allows the exploration of the structure of complex lipid structures in detail.^92^ Furthermore, the development of hybrid spectrometers such as the quadrupole‐time of flight (Q‐TOF) or the hybrid ion trap‐orbitrap spectrometers improves not only mass resolution and mass accuracy but also the detection sensitivity of lipids.^93^, ^94^ These technologies have been applied in lipid characterization of colorectal and prostate cancer‐derived exosomes with promising results.^17^, ^95^

**FIGURE 3:**
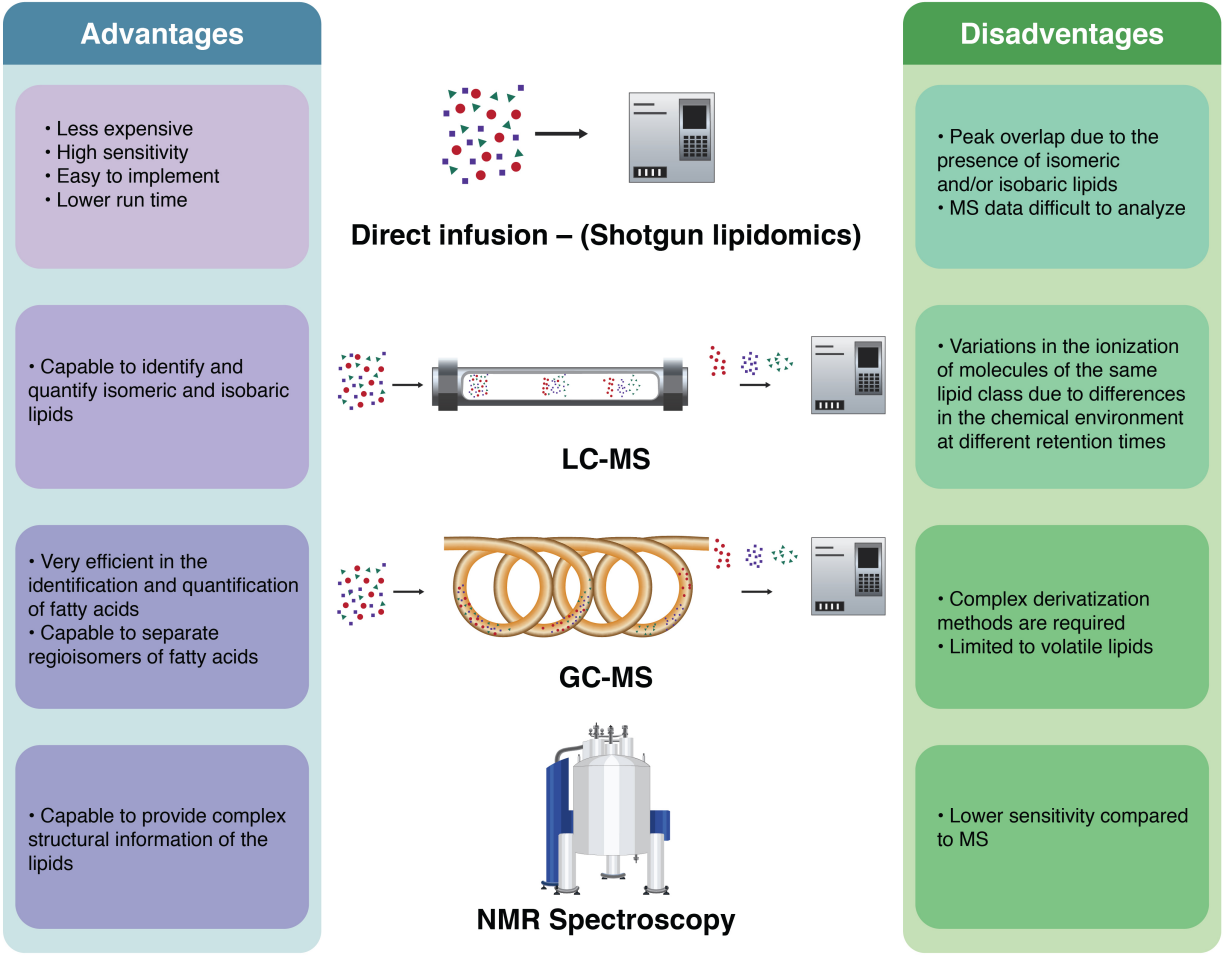
Relevant advantages and disadvantages of the most used analytical methods in lipidomic research. In LC‐MS and GC‐MS, analytes enter the MS detector as individual lipid species after chromatographic separation, overcoming some limitations observed in the direct infusion method. Despite the lower efficiency in lipid quantification observed in NMR spectroscopy, this method provides unique structural information about the lipid molecules

LC‐MS systems possess additional selectivity due to the appearance of different retention times as identification parameters, decreasing the complexity of the mass spectra, and improving the peak capacity of the sample.^96^ For lipidomic purposes, triple quadrupole instruments are still the most widely used systems in LC‐MS platforms.^97^ In this context, there is a wide variety of LC systems available for lipidomic analysis, each one with different advantages and disadvantages depending on the nature of the analyte. Recent advances in this field include the use of supercritical fluid LC to analyze plasma lipids with high throughput, high resolution and good reproducibility, resulting in reduced misidentification and enhanced data analysis.^98^ In exosome lipidomics, hyphenated micro‐LC‐Q‐TOF‐MS, as well as nanoflow ultrahigh‐performance liquid chromatography (UHPLC)‐MS/MS systems, have been used to study lipid alterations in exosomes from both urine samples and cell culture media, respectively, obtaining high‐quality results.^99^, ^100^ Furthermore, UHPLC‐MS and ultrahigh‐performance supercritical fluid chromatography (UHPSFC)‐MS provided high‐separation efficiency and short analysis times in a lipidomic study of human plasma‐derived exosomes.^23^ In this line, it was reported that using ammonium acetate and ammonium formate without acidifiers as mobile‐phase modifier systems in ESI (+) and ESI (−) modes, respectively, of UHPLC‐MS, increased the lipidome coverage in both lipid standard mixtures as well as in blood plasma lipids.^101^ Besides, the use of in silico simulations of data acquisition performance has been proposed to identify the optimal method parameters in LC‐MS/MS for lipidomic research, considering the synergistic relationship between MS method parameters and avoiding sub‐optimal results.^102^ Figure 3 summarizes some relevant advantages and disadvantages of MS and NMR technologies in lipidomic research.

### 5.2 Data processing and bioinformatics

The growing interest in lipidomics as a tool for the evaluation of cell homeostasis demands the development of bioinformatic workflows to identify, quantify and study the influence of lipids on metabolism. However, despite the existence of bioinformatic mechanisms for these purposes, some of them lack simplicity and interconnectivity and are not user‐friendly.^103^ Thus, the “Lipidomics Informatics for Life‐Science” platform has recently provided its lipidomics software tools with integrative and user‐friendly web interfaces. These tools include “LipidXplorer” for shotgun lipidomics,^104^ “Skyline for Lipidomics” to assemble targeted mass spectrometry methods for complex lipids,^105^ “LUX Score” for the quantification of systematic differences in the lipid composition of a lipidome,^106^ and “LipidHome”, to bridge the gap between theoretically identified lipid molecules and metadata.^107^ All these tools have been recently used to identify exosome lipidic biomarkers in pancreatic cancer.^11^

A similar bioinformatic platform extensively used to analyze exosome lipidomics data obtained by LC‐MS is the LIPID MAPS consortium. This system provides online tools to predict lipid structures from MS data, assigning lipid chemical structures, the corresponding systematic names and ontological information.^108^ This platform also contains LipidFinder, an openly available bioinformatic tool designed to curate MS data from lipidomics research by eliminating non‐lipid artifacts and reducing mistakes in the interpretation of MS spectra.^109^, ^110^ In exosome lipidomics, LIPID MAPS databases are being used to identify lipid perturbations in exosomes isolated from plasma of colorectal cancer individuals and in urinary exosomes from renal cell carcinoma patients, just to mention some examples.^99^, ^111^ Similar bioinformatics tools applied in exosome lipidomics include Lipid Profiler,^94^ MultiQuant,^10^ MetaboAnalyst,^12^ among others.

## 6 NOVEL EXOSOMAL LIPID‐BASED BIOMARKERS

**FIGURE 4:**
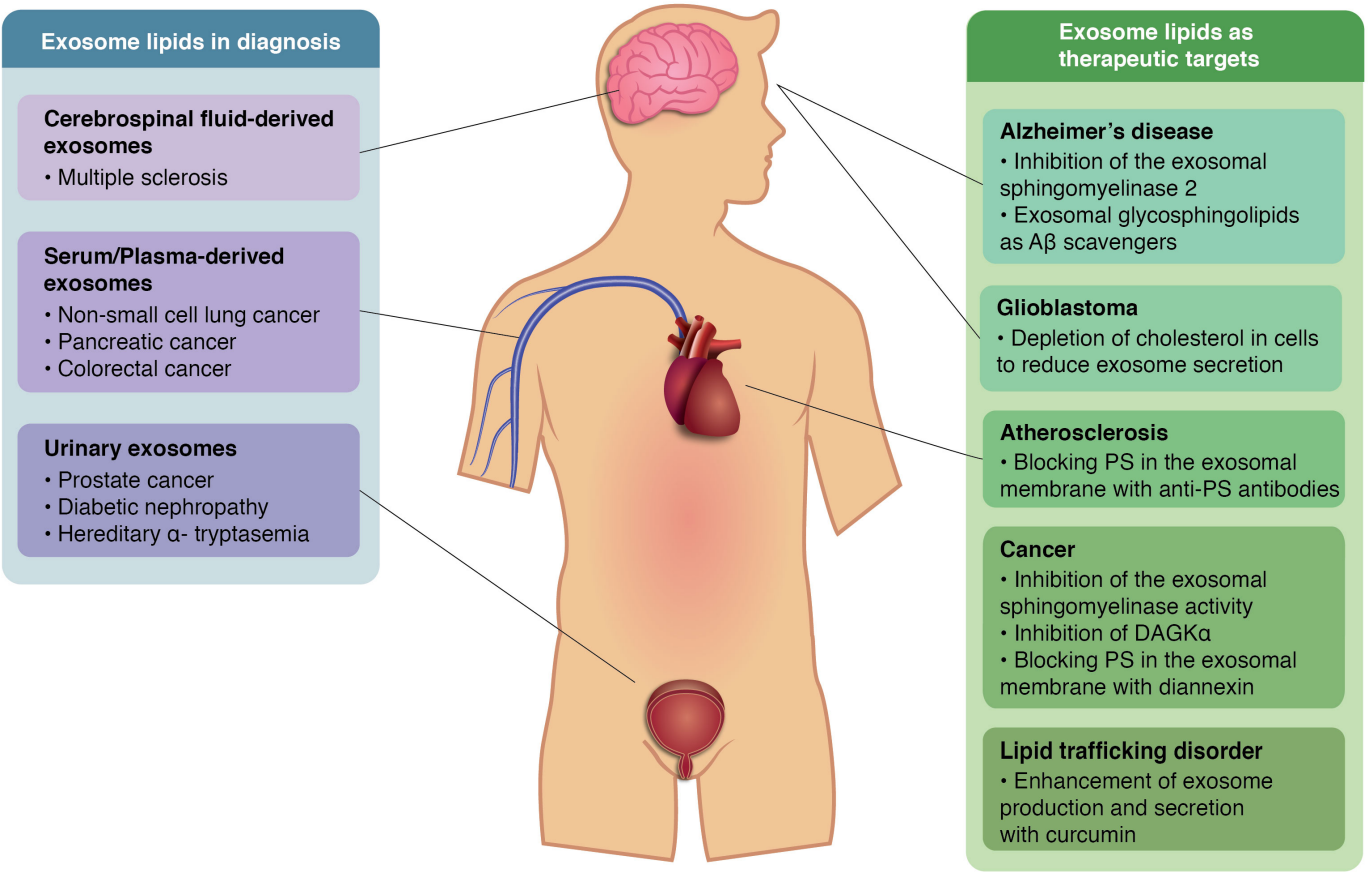
Summary of recent applications of exosomal lipids from different body fluids in biomedicine. The blue box shows the diseases in which exosomal lipids appear to be good candidates as diagnosis molecules classified by the source body fluid. The green box illustrates some disorders in which exosome lipids could act as therapeutic targets to control the disease progression. Aβ: amyloid‐β; PS: phosphatidylserine; DAGKα: diacylglycerol kinase α

Exosomes isolated from different body fluids represent a novel source of biomarkers for several diseases, including cancer (Figure 4). As the exosomal components derive from the exosome‐producer cell, these components evince the biological state of the cell and could potentially carry information about the health state of an organ or a tissue.^112^ Furthermore, it is reported that the signal‐to‐noise ratio is enhanced in exosomes compared to an unfractionated body fluid. In human plasma, the protein mass is dominated by a few proteins such as albumin and globulin, which would likely mask other low‐concentration biomarkers,^113^ obstructing their detection. In this sense, exosomes appear to be an interesting option to overcome these limitations. However, it is reported that the state‐of‐the‐art methods for EVs isolation from blood plasma results in the co‐purification of low‐density lipoproteins, obstructing further analysis and leading to data misinterpretation.^114^ Different studies, like the one by Boer et al.,^115^ have presented the development of multistep processes (i.e., ultracentrifugation, density gradient purification and size exclusion chromatography) to avoid lipoprotein contamination in EVs preparations from blood plasma with promising results. Nonetheless, the development of standardized and efficient methods for high‐purity exosome (and their constitutive elements) isolation from biological fluids remains a challenge.^116^

As innovative methods for exosome isolation and purification emerge, several exosomal components, such as lipids, have been proposed as promising biomarkers in diagnosis. In cancer diagnosis, for example, PS (18:1/18:1), LacCer (d18:1/16:0) and PS (18:0/18:2) isolated from urinary exosomes allow the discrimination between prostate cancer patients and healthy controls with 93% sensitivity and 100% specificity.^10^ Similar research in the area includes the use of serum and blood plasma exosome lipids in pancreatic^11^ and non‐small cell lung cancer diagnosis,^13^ as described in detail in Table 3.

**TABLE 3:** Relevant exosome lipids proposed as biomarkers for diagnosis

Disease	EVs source	Analytical method	Lipid biomarkers	Remarks	References
Prostate cancer	Urine	Hybrid triple quadrupole/linear ion trap mass spectrometer	LacCer (d18:1/16:0), PS (18:1/18:1) and PS (16:0–18:1).	93% sensitivity and 100% specificity by using the combination of the three lipids.	^10^
Non‐small cell lung cancer	Blood plasma	Ultra‐high‐resolution Fourier transform mass spectrometry	PC (18:1/18:2), PC (18:0/20:3), TG (54:6)	These three lipids were overlapped between two multivariate statistical methods: The Random Forest and the Least Absolute Shrinkage and Selection Operator.	^13^
Pancreatic cancer	Serum	UHPLC‐data‐dependent acquisition ‐MS	LPC (22:0), PC (P‐14:0/22:2) and PE (16:0/18:1) associated with tumor stage. PE (16:0/18:1) associated with patient overall survival.	This study proposes lipids associated with disease stage, tumor size, and patient overall survival.	^11^
Colorectal cancer	Serum	Quadrupole Time‐of‐Flight Mass Spectrometry	56 lipids (glycolipids, phospholipids, fatty acids and sphingolipids).	The joint pathway analysis revealed that sphingolipid and glycerophospholipid metabolisms exert the strongest discriminative power.	^111^
Diabetic nephropathy	Urine	UHPLC‐high‐resolution MS	PC, LPC, PIP2, DG and GM3	These lipids are differentially expressed in exosomes from diabetic and diabetic nephropathy patients.	^12^
Multiple sclerosis	Cerebrospinal fluid	HPLC‐Quattro Ultima Pt ESI tandem quadrupole mass spectrometer	SM	SM converted to Cer by acid sphingomyelinase with neurodegenerative effects.	^70^
Hereditary α‐tryptasemia	Urine	UHPLC‐Qtrap 6500	64 lipids (glycerophospholipids, glycerolipids and sterols)	The 64 lipids were significantly reduced in urinary exosomes of hereditary α‐tryptasemia patients.	^117^

Abbreviations: Cer, ceramide; DG, diglyceride; GM3, ganglioside; LacCer, Lactosyl ceramide; LPC, lysophosphatidylcholine; PC, phosphatidylcholine; PE, phosphatidylethanolamine; PIP2, phosphatidylinositol bisphosphate; PS, phosphatidylserine; SM, sphingomyelin.

Besides cancer, other disorders can be detected in body fluids through exosome lipids. Thus, urinary exosome lipids from diabetes mellitus and diabetic nephropathy patients have been studied. It was found that DG, TG, GM3 and LPC lipids were increased in diabetic nephropathy samples while exosomes derived from diabetic individuals were enriched in phosphatidylinositol bisphosphate (PIP2) and PC.^12^ Furthermore, exosome lipids from urine revealed that hereditary α‐tryptasemia patients produce exosomes reduced in glycerophospholipids, glycerolipids and sterols.^117^ Similarly, exosomes from the cerebrospinal fluid of multiple sclerosis patients are decreased in SM due to the overexpression of acid sphingomyelinase with neurodegenerative effects, generating new opportunities for the diagnosis and treatment of this disease.^70^

In the context of the COVID‐19 pandemic, exosomes appear to be important regulators of disease progression. Apparently, SARS‐CoV‐2 infection regulates exosome composition to express molecules that modulate inflammation, immune response and activation of the coagulation and complement pathways, contributing to tissue damage and organ failure.^118^ Furthermore, the lipidomic analysis revealed that exosomes isolated from the blood plasma of COVID‐19 patients are enriched in GM3 and SM and reduced in DAG.^119^ Thus, these lipids could represent promising alternatives to assess the progression and severity of the infection.

The development of a new method for isolating different populations of EVs based on their lipid affinity to cholera toxin B (CTB), annexin V (AV) and Shiga toxin B (STB),^120^ have brought the opportunity to develop more sensitive techniques for exosome‐based diagnosis.^112^ In this context, it was found that the fractionation of EVs by their binding affinity towards CTB, AV and STB, results in different EVs populations with different and unique protein compositions, and consequently, improved properties for the diagnosis of preeclampsia, high‐grade serous ovarian cancer, and head and neck squamous cell carcinoma.^121^, ^122^, ^123^ Since this method for the fractionation of EVs is based on their lipidic composition, future research in this field should focus on the enhancement of the diagnosis properties of exosome lipids after a CTB, AV and STB fractionation (see Table 3).

## 7 EXOSOME LIPIDS AS THERAPEUTIC TARGETS

Given the previously mentioned role of exosomes in the pathogenesis of some diseases, several strategies have been proposed to highlight their potential as novel therapeutic targets by inhibiting key aspects in their biology, such as biogenesis, release and cell uptake.^124^ These novel methods could be applied in disorders in which exosomes induce a pathological effect (Figure 4). For example, in cancer, it has been demonstrated that the amount of circulating EVs is correlated with cancer progression, and with the survival of patients with melanoma.^125^ In this case, a therapeutic intervention could be aimed at reducing the load of exosomes in blood by inhibiting their biogenesis or release.

One of the strategies proposed with this aim is the reduction of the endosomal sorting and exosome biogenesis through the inhibition of the sphingomyelinase, the enzyme that synthesizes ceramide from SM. This inhibition can be achieved with the blood‐pressure‐lowering drug amiloride, which has demonstrated an efficient in vivo reduction of the circulating tumor‐derived EVs with the subsequent reduction in tumor growth.^38^, ^126^ Similarly, the biosynthesis of ceramide has also been inhibited using GW4869 and some specific small interfering RNA, reducing exosome release.^127^, ^128^ However, a study in PC3 cells revealed that this interference in exosome biogenesis attained by inhibiting the synthesis of ceramide could be cell‐type specific, with lower or no effect in certain cell types.^129^

Similar lipid‐related molecules acting as therapeutic targets in exosomes include the diacylglycerol kinase α (DAGK α) and PS. In this sense, the inhibition of the DAGKα by the DAGK inhibitor II resulted in a decreased secretion of exosomes in J‐HM1‐2.2 cells.^130^ On their part, PS is a lipid molecule exposed on exosome surface important for cell adhesion.^131^ The evidence suggests that blocking PS with diannexin reduces the cellular uptake of exosomes, resulting also in a decreased growth of tumor xenografts in mice.^132^, ^133^ Furthermore, to prevent the exosome‐mediated cholesterol accumulation in atheroma‐associated cells, anti‐PS receptor antibodies were evaluated, resulting in a diminished internalization of exosomes in cells with favorable outcomes.^9^ However, therapeutic strategies based on blocking PS should be carefully designed to avoid interference with other physiological functions regulated by PS, such as the clearing of apoptotic cells.^124^

In brain astrocytes, it has been found that the amyloid‐β (Aβ) peptide stimulates the secretion of exosomes enriched in both ceramide and the ceramide‐sensitizer protein PAR‐4, with apoptotic effects. This deleterious effect was suppressed by inhibiting the activity of sphingomyelinase 2.^134^ Furthermore, exosomes can also act as Aβ scavengers by sequestering Aβ through the glycosphingolipids on the exosome surface.^135^ The inhibition of the sphingomyelinase 2 activity to avoid exosome‐induced apoptosis in astrocytes and the Aβ clearance effect of exogenous exosomes in the brain provides novel insights for therapeutic intervention in Alzheimer's disease.^136^, ^137^, ^138^

The cellular internalization of exosomes derived from glioblastoma cells involves the non‐classical, lipid raft‐dependent endocytosis negatively regulated by the lipid raft‐associated protein caveolin‐1 (CAV‐1).^139^ This study revealed that exosome internalization depends on the exosome‐induced phosphorylation of downstream targets of the lipid‐rafts‐associated extracellular signal‐regulated kinase‐1/2 (ERK1/2) and heat shock protein 27 (HSP27) under the negative regulation of CAV‐1. Consequently, exosome uptake seems to be significantly decreased after cholesterol depletion in recipient cells by exogenous statins or methyl‐β‐cyclodextrin. This evidence expands the current understanding of exosome uptake mechanisms and offers potential approaches to manipulate these processes by modulating the intracellular cholesterol levels in recipient cells.

For therapeutic purposes, not only strategies for inhibition of exosome biogenesis, release or uptake have been proposed, but also mechanisms to improve lipid trafficking by enhancing exosome production and release. In this sense, curcumin, a hydrophobic polyphenol, seems to increase exosome secretion in C6 glial cells by stimulating the synthesis of ceramide, thereby decreasing the lipid concentration in the endo‐lysosomal compartment.^140^ Therefore, this effect of curcumin over ceramide synthesis and exosome secretion could ameliorate the endo‐lysosomal lipid accumulation observed in lysosomal storage disorders.

## 8 CONCLUSIONS AND FUTURE PERSPECTIVES

This work provided an overview of lipids as critical molecules in exosome biology, not only as indispensable structural components but also as essential regulatory agents. These unique properties of exosomal lipids added to the intrinsic role of exosomes in intercellular communication and disease progression, stress the importance of these molecules to be considered as the new generation of exosomal components with potential biotechnological applications. In consequence, during the last few years, exosomal lipids have gained attention because of their potential use as biomarkers to develop non‐invasive diagnosis methods, and as therapeutic targets to control disease progression and pathogenesis. Furthermore, exosome lipidomics has emerged as a novel discipline to evaluate lipid alterations in exosomes under pathological conditions, and to understand the interactions and mechanisms by which these alterations modify cell communication pathways and induce pathogenesis. However, lipidomics, as a relatively new field in omic sciences, possess several limitations that should be solved to optimize the quality of the results.

First, standardized methods for sample preparation and storage need to be developed, especially considering the instability of lipids under certain temperatures,^141^ pH,^142^ or freezing conditions.^143^ Besides, conventional exosome isolation methods such as ultracentrifugation, polymer‐based precipitation, size exclusion, density gradient centrifugation and immunoaffinity capture, may induce loss of exosome integrity and co‐isolation of other non‐exosome EVs, disturbing the results of lipidomic analysis.^81^ Therefore, the optimization of these traditional methods for lipidomic studies or new exosome isolation systems is required. As an alternative, flow field‐flow fractionation has been proposed as a new size‐based isolation method with favorable outcomes in exosome lipidomics that needs to be further studied.^144^

Improvements in analytical methods to achieve full coverage of lipidomes are also required. Many isomeric/isobaric lipid species precludes the use of the shotgun approach in future lipidomic research. Therefore, innovative chromatographic separations need to be developed, like those previously proposed using mobile‐phase modifier systems.^101^ Similarly, migration from HPLC to 2.1 mm UHPLC or microflow LC systems may improve the sample throughput and the quality of the results.^83^

The absolute quantification of all lipid species in a lipidome remains a major challenge due to the limited number of commercially available lipid standards. Consequently, it is also necessary to establish a consensus in the field of lipidomics about how much accuracy is required to quantitate individual molecular lipid species. Moreover, structural validation of lipids is essential, especially in those proposed as biomarkers. In this sense, tandem MS analysis should be needed, as well as sample derivatization methods to validate functional groups.^145^

In summary, despite the recent achievements in the field of exosome lipidomics, research in this field is still incipient. Therefore, future comprehensive studies are indispensable to increase the current knowledge in exosome biology and their regulatory mechanisms, as well as the potential applications of exosomal lipids in biotechnology. Future research in this area should be performed by the hand of state‐of‐the‐art analytical procedures to overcome the limitations of current lipidomic studies and ensure high‐quality results, especially for clinical applications. Finally, translational research of novel exosome‐related technologies must emerge to propose solutions to a wide variety of health problems that currently lack efficient therapies and continue to affect millions of people around the world.

## CONFLICT OF INTEREST

The authors declare no conflict of interest.

## PEER REVIEW

The peer review history for this article is available at https://publons.com/publon/10.1111/tra.12803.

## References

[1] Akers JC, Gonda D, Kim R, Carter BS, Chen CC. Biogenesis of extracellular vesicles (EV): exosomes, microvesicles, retrovirus‐like vesicles, and apoptotic bodies. J Neuro‐Oncol. 2013;113(1):1‐11. 10.1007/s11060-013-1084-8. PMC5533094 23456661

[2] Delenclos M, Trendafilova T, Mahesh D, et al. Investigation of endocytic pathways for the internalization of exosome‐associated oligomeric alpha‐synuclein. Front Neurosci. 2017;11:172. 10.3389/fnins.2017.00172. 28424577 PMC5371652

[3] Johnstone RM, Adam M, Hammond JR, Orr L, Turbide C. Vesicle formation during reticulocyte maturation. Association of plasma membrane activities with released vesicles (exosomes). J Biol Chem. 1987;262(19):9412‐9420. 10.1016/S0021-9258(18)48095-7. 3597417

[4] Takahashi A et al. Exosomes maintain cellular homeostasis by excreting harmful DNA from cells. Nature Commun. 2017;8(1):15287. 10.1038/ncomms15287. 28508895 PMC5440838

[5] Théry C et al. Minimal information for studies of extracellular vesicles 2018 (MISEV2018): a position statement of the International Society for Extracellular Vesicles and update of the MISEV2014 guidelines. J Extracell Vesicles. 2018;7(1):1535750. 10.1080/20013078.2018.1535750. 30637094 PMC6322352

[6] Kang J‐S. The potential of exosomes as theragnostics in various clinical situations. Exosomes. 2020;2020:467‐486. 10.1016/B978-0-12-816053-4.00020-1.

[7] Cheng Y, Schorey JS. The function and therapeutic use of exosomes in bacterial infections. Exosomes. 2020;2020:123‐146. 10.1016/B978-0-12-816053-4.00006-7.

[8] Hong Y et al. Exosome as a vehicle for delivery of membrane protein therapeutics, PH20, for enhanced tumor penetration and antitumor efficacy. Adv Funct Mater. 2018;28(5):1703074. 10.1002/adfm.201703074.

[9] Zakharova L, Svetlova M, Fomina AF. T cell exosomes induce cholesterol accumulation in human monocytes via phosphatidylserine receptor. J Cell Physiol. 2007;212(1):174‐181. 10.1002/jcp.21013. 17299798

[10] Skotland T, Ekroos K, Kauhanen D, et al. Molecular lipid species in urinary exosomes as potential prostate cancer biomarkers. Eur J Cancer. 2017;70:122‐132. 10.1016/j.ejca.2016.10.011. 27914242

[11] Tao L et al. Metabolomics identifies serum and exosomes metabolite markers of pancreatic cancer. Metabolomics. 2019;15(6):86. 10.1007/s11306-019-1550-1. 31147790

[12] Kumari S, Singh A. Urinary exosomal lipidomics reveals markers for diabetic nephropathy. Curr Metabol. 2018;6:131‐139.

[13] Fan TWM, Zhang X, Wang C, et al. Exosomal lipids for classifying early and late stage non‐small cell lung cancer. Anal Chim Acta. 2018;1037:256‐264. 10.1016/j.aca.2018.02.051. 30292300 PMC6582997

[14] Choi DS, Kim DK, Kim YK, Gho YS. Proteomics, transcriptomics and lipidomics of exosomes and ectosomes. Proteomics. 2013;13:1554‐1571. 10.1002/pmic.201200329. 23401200

[15] Zhang H, Freitas D, Kim HS, et al. Identification of distinct nanoparticles and subsets of extracellular vesicles by asymmetric flow field‐flow fractionation. Nat Cell Biol. 2018;20:332‐343. 10.1038/s41556-018-0040-4. 29459780 PMC5931706

[16] Carayon K, Chaoui K, Ronzier E, et al. Proteolipidic composition of exosomes changes during reticulocyte maturation. J Biol Chem. 2011;286(39):34426‐34439. 10.1074/jbc.M111.257444. 21828046 PMC3190795

[17] Llorente A, Skotland T, Sylvänne T, et al. Molecular lipidomics of exosomes released by PC‐3 prostate cancer cells. Biochim Biophys Acta. 2013;1831:1302‐1309. 10.1016/j.bbalip.2013.04.011. 24046871

[18] Beloribi S et al. Exosomal lipids impact notch signaling and induce death of human pancreatic Tumoral SOJ‐6 cells. PLoS One. 2012;7(10):e47480. 10.1371/journal.pone.0047480. 23094054 PMC3477155

[19] Phuyal S, Skotland T, Hessvik NP, et al. The ether lipid precursor hexadecylglycerol stimulates the release and changes the composition of exosomes derived from PC‐3 cells. J Biol Chem. 2015;290(7):4225‐4237. 10.1074/jbc.M114.593962. 25519911 PMC4326831

[20] Hirsova P, Ibrahim SH, Krishnan A, et al. Lipid‐induced signaling causes release of inflammatory extracellular vesicles from hepatocytes. Gastroenterology. 2016;150:956‐967. 10.1053/j.gastro.2015.12.037. 26764184 PMC4808464

[21] Showalter MR, Wancewicz B, Fiehn O, et al. Primed mesenchymal stem cells package exosomes with metabolites associated with immunomodulation. Biochem Biophys Res Commun. 2019;512:729‐735. 10.1016/j.bbrc.2019.03.119. 30926165 PMC6682414

[22] Subra C, Laulagnier K, Perret B, Record M. Exosome lipidomics unravels lipid sorting at the level of multivesicular bodies. Biochimie. 2007;89:205‐212. 10.1016/j.biochi.2006.10.014. 17157973

[23] Peterka O et al. Lipidomic characterization of exosomes isolated from human plasma using various mass spectrometry techniques. Biochim Biophys Acta. 2020;1865(5):158634. 10.1016/j.bbalip.2020.158634. 31978556

[24] Wubbolts R, Leckie RS, Veenhuizen PTM, et al. Proteomic and biochemical analyses of human B cell‐derived exosomes: potential implications for their function and multivesicular body formation. J Biol Chem. 2003;278:10963‐10972. 10.1074/jbc.M207550200. 12519789

[25] Segawa K, Kurata S, Yanagihashi Y, Brummelkamp TR, Matsuda F, and Nagata S. Caspase‐mediated cleavage of phospholipid flippase for apoptotic phosphatidylserine exposure. Science. 2014;344(6188):1164‐1168. 10.1126/science.1252809. 24904167

[26] Miyanishi M, Tada K, Koike M, Uchiyama Y, Kitamura T, Nagata S. Identification of Tim4 as a phosphatidylserine receptor. Nature. 2007;450:435‐439. 10.1038/nature06307. 17960135

[27] Lea J, Sharma R, Yang F, Zhu H, Sally Ward E, Schroit AJ. Detection of phosphatidylserine‐positive exosomes as a diagnostic marker for ovarian malignancies: a proof of concept study. Oncotarget. 2017;8(9):14395‐14407. 10.18632/oncotarget.14795. 28122335 PMC5362413

[28] Kang YT et al. Isolation and profiling of circulating tumor‐associated exosomes using extracellular vesicular lipid–protein binding affinity based microfluidic device. Small. 2019;15(47):1903600. 10.1002/smll.201903600. PMC6951813 31588683

[29] Laulagnier K et al. Mast cell‐ and dendritic cell‐derived exosomes display a specific lipid composition and an unusual membrane organization. Biochem J. 2004;380(1):161‐171. 10.1042/bj20031594. 14965343 PMC1224152

[30] Colombo M et al. Analysis of ESCRT functions in exosome biogenesis, composition and secretion s the heterogeneity of extracellular vesicles. J Cell Sci. 2013;126(24):5553‐5565. 10.1242/jcs.128868. 24105262

[31] Skotland T, Hessvik NP, Sandvig K, Llorente A. Exosomal lipid composition and the role of ether lipids and phosphoinositides in exosome biology. J Lipid Res. 2019;60(1):9‐18. 10.1194/jlr.R084343. 30076207 PMC6314266

[32] Luo P et al. Metabolic characteristics of large and small extracellular vesicles from pleural effusion reveal biomarker candidates for the diagnosis of tuberculosis and malignancy. J Extracell Vesicles. 2020;9(1):1790158. 10.1080/20013078.2020.1790158. 32944177 PMC7480510

[33] Laulagnier K, Vincent‐Schneider H, Hamdi S, Subra C, Lankar D, Record M. Characterization of exosome subpopulations from RBL‐2H3 cells using fluorescent lipids. Blood Cells Mol Dis. 2005;35:116‐121. 10.1016/j.bcmd.2005.05.010. 16023874

[34] Singhto N, Vinaiphat A, Thongboonkerd V. Discrimination of urinary exosomes from microvesicles by lipidomics using thin layer liquid chromatography (TLC) coupled with MALDI‐TOF mass spectrometry. Sci Rep. 2019;9:13834. 10.1038/s41598-019-50195-z. 31554842 PMC6761130

[35] Lai RC, Lim SK. Membrane lipids define small extracellular vesicle subtypes secreted by mesenchymal stromal cells. J Lipid Res. 2019;60:318‐322. 10.1194/jlr.R087411. 30154233 PMC6358289

[36] Durcin M et al. Characterisation of adipocyte‐derived extracellular vesicle subtypes identifies distinct protein and lipid signatures for large and small extracellular vesicles. J Extracell Vesicles. 2017;6(1):1305677. 10.1080/20013078.2017.1305677. 28473884 PMC5405565

[37] Haraszti RA et al. High‐resolution proteomic and lipidomic analysis of exosomes and microvesicles from different cell sources. J Extracell Vesicles. 2016;5(1):32570. 10.3402/jev.v5.32570. 27863537 PMC5116062

[38] Trajkovic K, Hsu C, Chiantia S, et al. Ceramide triggers budding of exosome vesicles into multivesicular endosomes. Science. 2008;319(5867):1244‐1247. 10.1126/science.1153124. 18309083

[39] Boura E, Ivanov V, Carlson L‐A, Mizuuchi K, Hurley JH. Endosomal sorting complex required for transport (ESCRT) complexes induce phase‐separated microdomains in supported lipid bilayers. J Biol Chem. 2012;287(33):28144‐28151. 10.1074/jbc.M112.378646. 22718754 PMC3431677

[40] Zhao Z et al. Cholesterol impairs hepatocyte lysosomal function causing M1 polarization of macrophages via exosomal miR‐122‐5p. Exp Cell Res. 2020;387(1):111738. 10.1016/j.yexcr.2019.111738. 31759057

[41] Kulshreshtha A, Singh S, Ahmad M, et al. Simvastatin mediates inhibition of exosome synthesis, localization and secretion via multicomponent interventions. Sci Rep. 2019;9:16373. 10.1038/s41598-019-52765-7. 31704996 PMC6841733

[42] Stuffers S, Sem Wegner C, Stenmark H, Brech A. Multivesicular endosome biogenesis in the absence of ESCRTs. Traffic. 2009;10(7):925‐937. 10.1111/j.1600-0854.2009.00920.x. 19490536

[43] Holopainen JM, Angelova MI, Kinnunen PKJ. Vectorial budding of vesicles by asymmetrical enzymatic formation of ceramide in giant liposomes. Biophys J. 2000;78(2):830‐838. 10.1016/S0006-3495(00)76640-9. 10653795 PMC1300685

[44] Kajimoto T, Okada T, Miya S, Zhang L, Nakamura SI. Ongoing activation of sphingosine 1‐phosphate receptors mediates maturation of exosomal multivesicular endosomes. Nat Commun. 2013;4:2712. 10.1038/ncomms3712. 24231649

[45] Kooijman EE et al. Spontaneous curvature of phosphatidic acid and lysophosphatidic acid. Biochemistry. 2005;44(6):2097‐2102. 10.1021/bi0478502. 15697235

[46] Kooijman EE, Chupin V, de Kruijff B, Burger KNJ. Modulation of membrane curvature by phosphatidic acid and lysophosphatidic acid. Traffic. 2003;4(3):162‐174. 10.1034/j.1600-0854.2003.00086.x. 12656989

[47] Tanguy E, Kassas N, Vitale N. Protein–phospholipid interaction motifs: a focus on phosphatidic acid. Biomolecules. 2018;8(2):20. 10.3390/biom8020020. PMC6022864 29690573

[48] Ghossoub R, Lembo F, Rubio A, et al. Syntenin‐ALIX exosome biogenesis and budding into multivesicular bodies are controlled by ARF6 and PLD2. Nat Commun. 2014;5:3477. 10.1038/ncomms4477. 24637612

[49] Wu BX, Clarke CJ, Matmati N, Montefusco D, Bartke N, Hannun YA. Identification of novel anionic phospholipid binding domains in neutral sphingomyelinase 2 with selective binding preference. J Biol Chem. 2011;286:22362‐22371. 10.1074/jbc.M110.156471. 21550973 PMC3121384

[50] Kameoka S, Adachi Y, Okamoto K, Iijima M, Sesaki H. Phosphatidic acid and cardiolipin coordinate mitochondrial dynamics. Trends Cell Biol. 2018;28(1):67‐76. 10.1016/j.tcb.2017.08.011. 28911913 PMC5742555

[51] Rocha N et al. Cholesterol sensor ORP1L contacts the ER protein VAP to control Rab7–RILP–p150Glued and late endosome positioning. J Cell Biol. 2009;185(7):1209‐1225. 10.1083/jcb.200811005. 19564404 PMC2712958

[52] Sprong H, van der Sluijs P, van Meer G. How proteins move lipids and lipids move proteins. Nat Rev Mol Cell Biol. 2001;2(7):504‐513. 10.1038/35080071. 11433364

[53] Morel E et al. Phosphatidylinositol‐3‐phosphate regulates sorting and processing of amyloid precursor protein through the endosomal system. Nat Commun. 2013;4(1):2250. 10.1038/ncomms3250. 23907271 PMC3905799

[54] Raiborg C, Schink KO, Stenmark H. Class III phosphatidylinositol 3‐kinase and its catalytic product PtdIns3P in regulation of endocytic membrane traffic. FEBS J. 2013;280(12):2730‐2742. 10.1111/febs.12116. 23289851

[55] Kirkegaard T et al. Hsp70 stabilizes lysosomes and reverts Niemann–pick disease‐associated lysosomal pathology. Nature. 2010;463(7280):549‐553. 10.1038/nature08710. 20111001

[56] Matsuo H. Role of LBPA and Alix in multivesicular liposome formation and endosome organization. Science. 2004;303(5657):531‐534. 10.1126/science.1092425. 14739459

[57] Hessvik NP, Øverbye A, Brech A, et al. PIKfyve inhibition increases exosome release and induces secretory autophagy. Cell Mol Life Sci. 2016;73:4717‐4737. 10.1007/s00018-016-2309-8. 27438886 PMC11108566

[58] Egea‐Jimenez AL, Zimmermann P. Lipids in exosome biology. In: Gomez‐Cambronero J, Frohman MA, eds. Lipid Signaling in Human Diseases. Cham: Springer International Publishing; 2020:309‐336. 10.1007/164_2019_220 31087193

[59] Subra C et al. Exosomes account for vesicle‐mediated transcellular transport of activatable phospholipases and prostaglandins. J Lipid Res. 2010;51(8):2105‐2120. 10.1194/jlr.M003657. 20424270 PMC2903822

[60] Zarini S, Gijon MA, Ransome AE, Murphy RC, Sala A. Transcellular biosynthesis of cysteinyl leukotrienes in vivo during mouse peritoneal inflammation. Proc Natl Acad Sci USA. 2009;106(20):8296‐8301. 10.1073/pnas.0903851106. 19416808 PMC2688893

[61] Menon D et al. Lipid sensing by mTOR complexes via de novo synthesis of phosphatidic acid. J Biol Chem. 2017;292(15):6303‐6311. 10.1074/jbc.M116.772988. 28223357 PMC5391759

[62] Radu CG, Yang LV, Riedinger M, Au M, Witte ON. T cell chemotaxis to lysophosphatidylcholine through the G2A receptor. Proc Natl Acad Sci U S A. 2004;101:245‐250. 10.1073/pnas.2536801100. 14681556 PMC314170

[63] Perrin‐Cocon L, Agaugué S, Coutant F, et al. Secretory phospholipase A2 induces dendritic cell maturation. Eur J Immunol. 2004;34:2293‐2302. 10.1002/eji.200324797. 15259027 PMC2755771

[64] Blackwood RA et al. Phospholipase D activity facilitates Ca2+‐induced aggregation and fusion of complex liposomes. Am J Physiol‐Cell Physiol. 1997;272(4):C1279‐C1285. 10.1152/ajpcell.1997.272.4.C1279. 9142853

[65] Kobayashi T, Beuchat MH, Chevallier J, et al. Separation and characterization of late endosomal membrane domains. J Biol Chem. 2002;283:34384‐34392. 10.1074/jbc.M202838200. 12065580

[66] Hough KP, Wilson LS, Trevor JL, et al. Unique lipid signatures of extracellular vesicles from the airways of asthmatics. Sci Rep. 2018;8:10340. 10.1038/s41598-018-28655-9. 29985427 PMC6037776

[67] Hough KP et al. Exosomal transfer of mitochondria from airway myeloid‐derived regulatory cells to T cells. Redox Biol. 2018;18:54‐64. 10.1016/j.redox.2018.06.009. 29986209 PMC6031096

[68] Aswad H, Forterre A, Wiklander OPB, et al. Exosomes participate in the alteration of muscle homeostasis during lipid‐induced insulin resistance in mice. Diabetologia. 2014;57:2155‐2164. 10.1007/s00125-014-3337-2. 25073444 PMC4153976

[69] Olsen ASB, Færgeman NJ. Sphingolipids: membrane microdomains in brain development, function and neurological diseases. Open Biol. 2017;7:170069. 10.1098/rsob.170069. 28566300 PMC5451547

[70] Pieragostino D, Cicalini I, Lanuti P, et al. Enhanced release of acid sphingomyelinase‐enriched exosomes generates a lipidomics signature in CSF of multiple sclerosis patients. Sci Rep. 2018;8:3071. 10.1038/s41598-018-21497-5. 29449691 PMC5814401

[71] Osenkowski P, Ye W, Wang R, Wolfe MS, Selkoe DJ. Direct and potent regulation of γ‐secretase by its lipid microenvironment. J Biol Chem. 2008;283:22529‐22540. 10.1074/jbc.M801925200. 18539594 PMC2504869

[72] Beloribi‐Djefaflia S, Siret C, Lombardo D. Exosomal lipids induce human pancreatic tumoral MiaPaCa‐2 cells resistance through the CXCR4‐SDF‐1α signaling axis. Oncoscience. 2015;2(1):15‐30. 10.18632/oncoscience.96. 25821841 PMC4341461

[73] Crewe C et al. An endothelial‐to‐adipocyte extracellular vesicle Axis governed by metabolic state. Cell. 2018;175(3):695‐708. 10.1016/j.cell.2018.09.005. 30293865 PMC6195477

[74] Flaherty SE, Grijalva A, Xu X, Ables E, Nomani A, Ferrante AW. A lipase‐independent pathway of lipid release and immune modulation by adipocytes. Science. 2019;363(6430):989‐993. 10.1126/science.aaw2586. 30819964 PMC6579605

[75] Buratta S et al. Lipotoxic stress alters the membrane lipid profile of extracellular vesicles released by Huh‐7 hepatocarcinoma cells. Sci Rep. 2021;11(1):4613. 10.1038/s41598-021-84268-9. 33633289 PMC7907093

[76] Liao C‐Y, Song MJ, Gao Y, Mauer AS, Revzin A, Malhi H. Hepatocyte‐derived Lipotoxic extracellular vesicle sphingosine 1‐phosphate induces macrophage chemotaxis. Front Immunol. 2018;9:2980. 10.3389/fimmu.2018.02980. 30619336 PMC6305739

[77] Barry OP, Praticò D, Lawson JA, FitzGerald GA. Transcellular activation of platelets and endothelial cells by bioactive lipids in platelet microparticles. J Clin Investig. 1997;99:2118‐2127. 10.1172/JCI119385. 9151784 PMC508042

[78] Xiang X, Poliakov A, Liu C, et al. Induction of myeloid‐derived suppressor cells by tumor exosomes. Int J Cancer. 2009;124:2621‐2633. 10.1002/ijc.24249. 19235923 PMC2757307

[79] Deng Z, Mu J, Tseng M, et al. Enterobacteria‐secreted particles induce production of exosome‐like S1P‐containing particles by intestinal epithelium to drive Th17‐mediated tumorigenesis. Nat Commun. 2015;6:6956. 10.1038/ncomms7956. 25907800 PMC4410277

[80] Coakley G, Wright MD, Borger JG. *Schistosoma mansoni*‐derived lipids in extracellular vesicles: potential agonists for eosinophillic tissue repair. Front Immunol. 2019;10:1010. 10.3389/fimmu.2019.01010. 31134080 PMC6514238

[81] Rustam YH, Reid GE. Analytical challenges and recent advances in mass spectrometry based lipidomics. Anal Chem. 2018;90:374‐397. 10.1021/acs.analchem.7b04836. 29166560

[82] Han X. Lipidomics: Comprehensive Mass Spectrometry of Lipids. Hoboken, NJ, USA: Wiley; 2016.

[83] Cajka T, Fiehn O. Toward merging untargeted and targeted methods in mass spectrometry‐based metabolomics and lipidomics. Anal Chem. 2016;88:524‐545. 10.1021/acs.analchem.5b04491. 26637011

[84] Beger RD, Schnackenberg LK, Holland RD, Li D, Dragan Y. Metabonomic models of human pancreatic cancer using 1D proton NMR spectra of lipids in plasma. Metabolomics. 2006;2(3):125‐134. 10.1007/s11306-006-0026-2.

[85] Yang Y‐L, Chong C‐P, Tsai M‐H, Liu M‐Y. Analysis of in vitro oxidized human LDL phospholipids by solid‐phase extraction and micellar electrokinetic capillary chromatography. Biomed Chromatogr. 2012;26(4):441‐448. 10.1002/bmc.1684. 22392513

[86] Pearce JM, Komoroski RA, Mrak RE. Phospholipid composition of postmortem schizophrenic brain by 31 P NMR spectroscopy. Mag Reson Med. 2009;61(1):28‐34. 10.1002/mrm.21820. PMC2610237 19097198

[87] Oostendorp M, Engelke UF, Willemsen MA, Wevers RA. Diagnosing inborn errors of lipid metabolism with proton nuclear magnetic resonance spectroscopy. Clin Chem. 2006;52(7):1395‐1405. 10.1373/clinchem.2006.069112. 16709621

[88] Kostara CE, Papathanasiou A, Cung MT, Elisaf MS, Goudevenos J, Bairaktari ET. Evaluation of established coronary heart disease on the basis of HDL and non‐HDL NMR lipid profiling. J Proteome Res. 2010;9(2):897‐911. 10.1021/pr900783x. 20020777

[89] Jafari M, Kadivar M, Keramat J. Detection of adulteration in Iranian olive oils using instrumental (GC, NMR, DSC) methods. J Am Oil Chem Soc. 2009;86(2):103‐110. 10.1007/s11746-008-1333-8.

[90] García‐González DL, Mannina L, D'Imperio M, Segre AL, Aparicio R. Using 1H and 13C NMR techniques and artificial neural networks to detect the adulteration of olive oil with hazelnut oil. Eur Food Res Technol. 2004;219(5):545‐548. 10.1007/s00217-004-0996-0.

[91] Barison A, Pereira da Silva CW, Campos FR, Simonelli F, Lenz CA, Ferreira AG. A simple methodology for the determination of fatty acid composition in edible oils through 1H NMR spectroscopy. Magn Reson Chem. 2010;48(8):642‐650. 10.1002/mrc.2629. 20589730

[92] Lydic TA, Busik JV, Esselman WJ, Reid GE. Complementary precursor ion and neutral loss scan mode tandem mass spectrometry for the analysis of glycerophosphatidylethanolamine lipids from whole rat retina. Anal Bioanal Chem. 2009;394(1):267‐275. 10.1007/s00216-009-2717-9. 19277613 PMC4112091

[93] Chernushevich IV, Loboda AV, Thomson BA. An introduction to quadrupole‐time‐of‐flight mass spectrometry. J Mass Spectrom. 2001;36:849‐865. 10.1002/jms.207. 11523084

[94] Ejsing CS, Moehring T, Bahr U, et al. Collision‐induced dissociation pathways of yeast sphingolipids and their molecular profiling in total lipid extracts: a study by quadrupole TOF and linear ion trap‐orbitrap mass spectrometry. J Mass Spectrom. 2006;41:372‐389. 10.1002/jms.997. 16498600

[95] Lydic TA, Townsend S, Adda CG, Collins C, Mathivanan S, Reid GE. Rapid and comprehensive ‘shotgun’ lipidome profiling of colorectal cancer cell derived exosomes. Methods. 2015;87:83‐95. 10.1016/j.ymeth.2015.04.014. 25907253 PMC4615275

[96] Triebl A, Hartler J, Trötzmüller M, Köfeler HC. Lipidomics: prospects from a technological perspective. Biochim Biophys Acta. 2017;1862:740‐746. 10.1016/j.bbalip.2017.03.004. PMC6013030 28341148

[97] Holčapek M, Jirásko R, Lísa M. Recent developments in liquid chromatography‐mass spectrometry and related techniques. J Chromatogr A. 2012;1259:3‐15. 10.1016/j.chroma.2012.08.072. 22959775

[98] Takeda H, Izumi Y, Takahashi M, et al. Widely‐targeted quantitative lipidomics method by supercritical fluid chromatography triple quadrupole mass spectrometry. J Lipid Res. 2018;59:1283‐1293. 10.1194/jlr.D083014. 29724780 PMC6027907

[99] del Boccio P, Raimondo F, Pieragostino D, et al. A hyphenated microLC‐Q‐TOF‐MS platform for exosomal lipidomics investigations: application to RCC urinary exosomes. Electrophoresis. 2012;33:689‐696. 10.1002/elps.201100375. 22451062

[100] Yang JS, Kim JY, Lee JC, Moon MH. Investigation of lipidomic perturbations in oxidatively stressed subcellular organelles and exosomes by asymmetrical flow field–flow fractionation and nanoflow ultrahigh performance liquid chromatography–tandem mass spectrometry. Anal Chim Acta. 2019;1073:79‐89. 10.1016/j.aca.2019.04.069. 31146839

[101] Cajka T, Fiehn O. Increasing lipidomic coverage by selecting optimal mobile‐phase modifiers in LC–MS of blood plasma. Metabolomics. 2016;12:34. 10.1007/s11306-015-0929-x.

[102] Hutchins PD, Russell JD, Coon JJ. Accelerating lipidomic method development through in silico simulation. Anal Chem. 2019;91:9698‐9706. 10.1021/acs.analchem.9b01234. 31298839 PMC6716604

[103] Schwudke D, Shevchenko A, Hoffmann N, Ahrends R. Lipidomics informatics for life‐science. J Biotechnol. 2017;261:131‐136. 10.1016/j.jbiotec.2017.08.010. 28822794

[104] Herzog R et al. Lipidxplorer: a software for consensual cross‐platform lipidomics. PLoS One. 2012;7(1):e29851. 10.1371/journal.pone.0029851. 22272252 PMC3260173

[105] Peng B, Ahrends R. Adaptation of skyline for targeted lipidomics. J Proteome Res. 2016;15:291‐301. 10.1021/acs.jproteome.5b00841. 26616352

[106] Marella C, Torda AE, Schwudke D. The LUX score: a metric for Lipidome homology. PLoS Comput Biol. 2015;11:e1004511. 10.1371/journal.pcbi.1004511. 26393792 PMC4578897

[107] Foster JM, Moreno P, Fabregat A, et al. LipidHome: a database of theoretical lipids optimized for high throughput mass spectrometry lipidomics. PLoS One. 2013;8:e61951. 10.1371/journal.pone.0061951. 23667450 PMC3646891

[108] Fahy E, Sud M, Cotter D, Subramaniam S. LIPID MAPS online tools for lipid research. Nucleic Acids Res. 2007;35:W606‐W612. 10.1093/nar/gkm324. 17584797 PMC1933166

[109] O'Connor A et al. LipidFinder: a computational workflow for discovery of lipids identifies eicosanoid‐phosphoinositides in platelets. JCI Insight. 2017;2(7):e91634. 10.1172/jci.insight.91634. 28405621 PMC5374061

[110] Fahy E, Alvarez‐Jarreta J, Brasher CJ, et al. LipidFinder on LIPID MAPS: peak filtering, MS searching and statistical analysis for lipidomics. Bioinformatics. 2019;35(4):685‐687. 10.1093/bioinformatics/bty679. 30101336 PMC6378932

[111] Eylem CC, Yilmaz M, Derkus B, et al. Untargeted multi‐omic analysis of colorectal cancer‐specific exosomes reveals joint pathways of colorectal cancer in both clinical samples and cell culture. Cancer Lett. 2020;469:186‐194. 10.1016/j.canlet.2019.10.038. 31669517

[112] Lai RC, Tan KH, Lim SK. Membrane lipid binding molecules for the isolation of bona fide extracellular vesicle types and associated biomarkers in liquid biopsy. J Cancer Meta Treat. 2019;5:65. 10.20517/2394-4722.2019.011.

[113] Hortin GL, Sviridov D, Anderson NL. High‐abundance polypeptides of the human plasma proteome comprising the top 4 logs of polypeptide abundance. Clin Chem. 2008;54:1608‐1616. 10.1373/clinchem.2008.108175. 18687737

[114] Sódar BW et al. Low‐density lipoprotein mimics blood plasma‐derived exosomes and microvesicles during isolation and detection. Sci Rep. 2016;6(1):24316. 10.1038/srep24316. 27087061 PMC4834552

[115] de Boer C et al. Analysis of the regenerative capacity of human serum exosomes after a simple multistep separation from lipoproteins. J Tissue Eng Regen Med. 2021;15(1):63‐77. 10.1002/term.3155. 33175463

[116] Ji X et al. A novel method of high‐purity extracellular vesicle enrichment from microliter‐scale human serum for proteomic analysis. Electrophoresis. 2021;42(3):245‐256. 10.1002/elps.202000223. 33169421 PMC8018574

[117] Glover SC, Nouri MZ, Tuna KM, et al. Lipidomic analysis of urinary exosomes from hereditary α‐tryptasemia patients and healthy volunteers. FASEB BioAdv. 2019;1:624‐638. 10.1096/fba.2019-00030. 31803861 PMC6892164

[118] Barberis E et al. Circulating exosomes are strongly involved in SARS‐CoV‐2 infection. Front Mol Biosci. 2021;8:632290. 10.3389/fmolb.2021.632290. 33693030 PMC7937875

[119] Song J‐W et al. Omics‐driven systems interrogation of metabolic dysregulation in COVID‐19 pathogenesis. Cell Metabolism. 2020;32(2):188‐202. 10.1016/j.cmet.2020.06.016. 32610096 PMC7311890

[120] Lai RC, Tan SS, Yeo RWY, et al. MSC secretes at least 3 EV types each with a unique permutation of membrane lipid, protein and RNA. J Extracell Vesicles. 2016;5:29828. 10.3402/jev.v5.29828. 26928672 PMC4770866

[121] Hian Tan K, Sim Tan S, Sze SK, Ryan Lee WK, Jack Ng M, Kiang Lim S. Plasma biomarker discovery in preeclampsia using a novel differential isolation technology for circulating extracellular vesicles. Am J Obstet Gynecol. 2014;211(4):380.e1‐380.e13. 10.1016/j.ajog.2014.03.038. 24657793

[122] Reiner AT, Tan S, Agreiter C, et al. EV‐associated MMP9 in high‐grade serous ovarian cancer is preferentially localized to Annexin V‐binding EVs. Dis Markers. 2017;2017:9653194. 10.1155/2017/9653194. 28607529 PMC5451862

[123] Rodrigues‐Junior DM, Tan SS, de Souza Viana L, et al. A preliminary investigation of circulating extracellular vesicles and biomarker discovery associated with treatment response in head and neck squamous cell carcinoma. BMC Cancer. 2019;19:373. 10.1186/s12885-019-5565-9. 31014274 PMC6480898

[124] el Andaloussi S, Mäger I, Breakefield XO, Wood MJA. Extracellular vesicles: biology and emerging therapeutic opportunities. Nat Rev Drug Discov. 2013;12:347‐357. 10.1038/nrd3978. 23584393

[125] Logozzi M, de Milito A, Lugini L, et al. High levels of exosomes expressing CD63 and caveolin‐1 in plasma of melanoma patients. PLoS One. 2009;4:e5219. 10.1371/journal.pone.0005219. 19381331 PMC2667632

[126] Bianco F, Perrotta C, Novellino L, et al. Acid sphingomyelinase activity triggers microparticle release from glial cells. EMBO J. 2009;28:1043‐1054. 10.1038/emboj.2009.45. 19300439 PMC2664656

[127] Kosaka N, Iguchi H, Yoshioka Y, Takeshita F, Matsuki Y, Ochiya T. Secretory mechanisms and intercellular transfer of microRNAs in living cells. J Biol Chem. 2010;285:17442‐17452. 10.1074/jbc.M110.107821. 20353945 PMC2878508

[128] Essandoh K, Yang L, Wang X, et al. Blockade of exosome generation with GW4869 dampens the sepsis‐induced inflammation and cardiac dysfunction. Biochim Biophys Acta. 2015;1852:2362‐2371. 10.1016/j.bbadis.2015.08.010. 26300484 PMC4581992

[129] Phuyal S, Hessvik NP, Skotland T, Sandvig K, Llorente A. Regulation of exosome release by glycosphingolipids and flotillins. FEBS J. 2014;281:2214‐2227. 10.1111/febs.12775. 24605801

[130] Alonso R, Mazzeo C, Rodriguez MC, et al. Diacylglycerol kinase α regulates the formation and polarisation of mature multivesicular bodies involved in the secretion of Fas ligand‐containing exosomes in T lymphocytes. Cell Death Differ. 2011;18:1161‐1173. 10.1038/cdd.2010.184. 21252909 PMC3131963

[131] Zagórska A, Través PG, Lew ED, Dransfield I, Lemke G. Diversification of TAM receptor tyrosine kinase function. Nat Immunol. 2014;15(10):920‐928. 10.1038/ni.2986. 25194421 PMC4169336

[132] Al‐Nedawi K, Meehan B, Kerbel RS, Allison AC, Rak A. Endothelial expression of autocrine VEGF upon the uptake of tumor‐derived microvesicles containing oncogenic EGFR. Proc Natl Acad Sci U S A. 2009;106:3794‐3799. 10.1073/pnas.0804543106. 19234131 PMC2656159

[133] Lima LG, Chammas R, Monteiro RQ, Moreira MEC, Barcinski MA. Tumor‐derived microvesicles modulate the establishment of metastatic melanoma in a phosphatidylserine‐dependent manner. Cancer Lett. 2009;283:168‐175. 10.1016/j.canlet.2009.03.041. 19401262

[134] Wang G, Dinkins M, He Q, et al. Astrocytes secrete exosomes enriched with proapoptotic ceramide and prostate apoptosis response 4 (PAR‐4): potential mechanism of apoptosis induction in Alzheimer disease (AD). J Biol Chem. 2012;287(25):21384‐21395. 10.1074/jbc.M112.340513. 22532571 PMC3375560

[135] Yuyama K, Sun H, Usuki S, et al. A potential function for neuronal exosomes: sequestering intracerebral amyloid‐β peptide. FEBS Lett. 2015;589:84‐88. 10.1016/j.febslet.2014.11.027. 25436414

[136] Tsunemi T, Hamada K, Krainc D. ATP13A2/PARK9 regulates secretion of exosomes and ‐synuclein. J Neurosci. 2014;34(46):15281‐15287. 10.1523/JNEUROSCI.1629-14.2014. 25392495 PMC4228131

[137] Kong SMY et al. Parkinson's disease‐linked human PARK9/ATP13A2 maintains zinc homeostasis and promotes α‐Synuclein externalization via exosomes. Hum Mol Genet. 2014;23(11):2816‐2833. 10.1093/hmg/ddu099. 24603074

[138] Chang C, Lang H, Geng N, Wang J, Li N, Wang X. Exosomes of BV‐2 cells induced by alpha‐synuclein: important mediator of neurodegeneration in PD. Neurosci Lett. 2013;548:190‐195. 10.1016/j.neulet.2013.06.009. 23792198

[139] Svensson KJ, Christianson HC, Wittrup A, et al. Exosome uptake depends on ERK1/2‐heat shock protein 27 signaling and lipid raft‐mediated endocytosis negatively regulated by caveolin‐1. J Biol Chem. 2013;288(24):17713‐17724. 10.1074/jbc.M112.445403. 23653359 PMC3682571

[140] García‐Seisdedos D, Babiy B, Lerma M, et al. Curcumin stimulates exosome/microvesicle release in an in vitro model of intracellular lipid accumulation by increasing ceramide synthesis. Biochim Biophys Acta. 1865;2020:158638. 10.1016/j.bbalip.2020.158638. 31988047

[141] Liebisch G, Drobnik W, Lieser B, Schmitz G. High‐throughput quantification of lysophosphatidylcholine by electrospray ionization tandem mass spectrometry. Clin Chem. 2002;48:2217‐2224. 10.1093/clinchem/48.12.2217. 12446479

[142] Scherer M, Schmitz G, Liebisch G. High‐throughput analysis of sphingosine 1‐phosphate, sphinganine 1‐phosphate, and lysophosphatidic acid in plasma samples by liquid chromatography: tandem mass spectrometry. Clin Chem. 2009;55:1218‐1222. 10.1373/clinchem.2008.113779. 19325012

[143] Kim J, Hoppel CL. Comprehensive approach to the quantitative analysis of mitochondrial phospholipids by HPLC‐MS. J Chromatogr B Analyt Technol Biomed Life Sci. 2013;912:105‐114. 10.1016/j.jchromb.2012.10.036. PMC4135309 23266842

[144] Yang JS, Lee JC, Byeon SK, Rha KH, Moon MH. Size dependent lipidomic analysis of urinary exosomes from patients with prostate cancer by flow field‐flow fractionation and nanoflow liquid chromatography‐tandem mass spectrometry. Anal Chem. 2017;89:2488‐2496. 10.1021/acs.analchem.6b04634. 28192938

[145] Wood PL, Cebak JE. Lipidomics biomarker studies: errors, limitations, and the future. Biochem Biophys Res Commun. 2018;504(7):569‐575. 10.1016/j.bbrc.2018.03.188. 29596837

